# Supplementary material for: Coordination-induced O-H/N-H bond weakening by a redox non-innocent, aluminum-containing radical
Source: Nat Commun. 2024 Feb 13;15:1315. doi: 10.1038/s41467-024-45721-1 (PMC10864259; doi:10.1038/s41467-024-45721-1)
Supplement: Supplementary file 1 — Supplementary Information [file 41467_2024_45721_MOESM1_ESM.pdf]

Supplementary Information for

Coordination-induced O-H/N-H bond weakening by a redox non-innocent, aluminum-containing radical

Soumen Sinhababu,[1] Roushan Prakash Singh,[1] Maxim R. Radzhabov,[1] Jugal Kumawat,[2] Daniel H. Ess,[2] Neal P. Mankad*[1]

[1]Department of Chemistry, University of Illinois Chicago, Chicago, IL 60607 (USA)
[2]Department of Chemistry and Biochemistry, Brigham Young University, Provo 84604, UT (USA)

*E-mail: npm@uic.edu

Table of Contents:

1. Experimental Section S2
2. NMR and IR spectra S8
3. Kinetic Experiments S21
4. Computations S23
5. References S26

1. Experimental Section

General Considerations. All manipulations were performed under a dry nitrogen atmosphere using either Schlenk line[1] or glovebox techniques. Solvents were dried using a Glass Contour Solvent System built by Pure Process Technology, LLC,[2] or purified by repeated freeze-pump thaw cycles followed by prolonged storage over activated, 3-Å molecular sieves. Literature procedure was followed in the synthesis of LAl(Me)Fp,[3] LAl(Me)I,[4] and NaW(Cp)$(CO)_3$.[5] Unless otherwise specified, all other chemicals were purchased from commercial sources and used without further purification.

^{1}H and ^{13}C{^{1}H} NMR spectra were recorded using Bruker Avance DPX-400 or DPX-500 MHz NMR spectrometers, and chemical shifts were referenced to the residual solvent peaks. FT-IR spectra were recorded on powder samples using a Bruker ALPHA spectrometer fitted with a diamond-ATR detection unit. Kinetics experiments were carried out in Bruker DRX 500 MHz. Elemental analyses were conducted by Atlantic Microlab in Norcross, GA (for **3**, **4**, **5**, **6c**) and Midwest Microlab, LLC, in Indianapolis, IN (for **2**, **6b**, **6d**). X-ray diffraction data collections were performed (for **3** and **5**) using a Bruker D8 QUEST ECO diffractometer under its default manufacturer settings. Crystals were covered with a cryoprotectant (paratone-n oil), transferred to a nylon loop, and cooled to 100 K. Data collection for **2** was carried out at Advanced Photon Source, Argonne National Laboratory, using a Bruker APEX-II CCD Detector, with a wavelength of 0.61991Å. Standard solution and refinement methods[6] within the SHELX package[7] were applied as specified in the supporting CIF files.

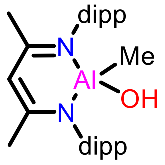

Synthesis of 6a: Inside the glove box, water (1.7 µL, 0.09 mmol) in diethyl ether (1 mL) was added dropwise to a solution of **1** (50 mg, 0.08 mmol) in toluene (5 mL) at -30 °C. After addition, the reaction mixture was stirred for 3 h at room temperature, and solvent was removed under reduced pressure to get an oily residue. The oily residue was dissolved in pentane (2 mL) and filtered through Celite. Toluene (0.2 mL) was added to the filtrate, and the solution was kept at -30 °C overnight. Red solids (Fp_2 formed from the remaining FpH) were separated from the colorless solution. Finally, evaporation to dryness yielded **6a** as a colorless solid. Yield: (30 mg, 82%). ^{1}H NMR (C_6D_6, 500 MHz): δ -0.88 (s, 3H, AlC*H*$_3$), 0.54 (s, 1H, AlO*H*), 1.08 (d, 6H, *J* = 7 Hz, CH(C*H*$_3$)$_2$), 1.22 (d, 6H, *J* = 7 Hz, CH(C*H*$_3$)$_2$), 1.33 (d, 12H, *J* = 7 Hz, CH(C*H*$_3$)$_2$), 1.57 (s, 6H, C*H*$_3$), 3.25 (sept, 2H, *J* = 6 Hz, C*H*(CH$_3$)$_2$), 3.70 (sept, 2H, *J* = 6 Hz, C*H*(CH$_3$)$_2$), 4.93 (s, 1H, *γ-CH*), 7.09 (t, 2H, *J* = 4 Hz, Ar), 7.16 (d, 3H, *J* = 4 Hz, Ar). ^{13}C{^{1}H}NMR (C_6D_6, 125 MHz): δ Signal for the CH_3Al group could not be observed, 23.3 (*C*H$_3$), 24.1 (CH(*C*H$_3$)$_2$), 24.4 (CH(*C*H$_3$)$_2$), 24.9 (CH(*C*H$_3$)$_2$), 26.3 (CH(*C*H$_3$)$_2$), 27.9 (*C*H(CH$_3$)$_2$), 29.0 (*C*H(CH$_3$)$_2$), 97.5 (*γ-C*), 124.0 (Ar), 124.9 (Ar), 127.3 (Ar), 140.8 (Ar), 143.5 (Ar), 145.5 (Ar), 169.4 (*C*N). These ^{1}H and ^{13}C NMR data match with a previous report.[8]

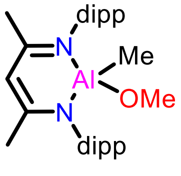

Synthesis of 6b: Inside the glove box, methanol (4 µL, 0.09 mmol) was added dropwise to a solution of **1** (50 mg, 0.08 mmol) in toluene (5 mL) at -30 °C. After addition, the reaction mixture was stirred for 3 h at room temperature, and solvent was removed under reduced pressure to get an oily residue. The oily residue was dissolved in pentane (2 mL) and filtered through Celite. Toluene (0.2 mL) was added to the filtrate, and the solution was kept at -30 °C overnight. Red solids (Fp_2

formed from the remaining FpH) were separated from the colorless solution. Finally, evaporation to dryness yielded **6b** as a colorless solid. Yield: (33 mg, 87%). ^{1}H NMR (C_6D_6, 500 MHz): δ -0.83 (s, 3H, AlC*H*$_3$), 1.08 (d, 6H, *J* = 7 Hz, CH(C*H*$_3$)$_2$), 1.26 (d, 6H, *J* = 7 Hz, CH(C*H*$_3$)$_2$), 1.30 (d, 6H, *J* = 7 Hz, CH(C*H*$_3$)$_2$), 1.46 (d, 6H, *J* = 7 Hz, CH(C*H*$_3$)$_2$), 1.57 (s, 6H, C*H*$_3$), 3.21 (sept, 2H, *J* = 6 Hz, C*H*(CH$_3$)$_2$), 3.66 (sept, 2H, *J* = 6 Hz, C*H*(CH$_3$)$_2$), 3.78 (s, 3H, O*Me*), 4.93 (s, 1H, *γ-CH*), 7.09 (d, 1H, *J* = 3 Hz, Ar), 7.10 (d, 1H, *J* = 3 Hz, Ar), 7.15-7.17 (m, 3H, Ar). ^{13}C{^{1}H}NMR (C_6D_6, 125 MHz): δ -14.9 (AlCH$_3$), 23.0 (*C*H$_3$), 24.2 (CH(*C*H$_3$)$_2$), 24.3 (CH(*C*H$_3$)$_2$), 24.5 (CH(*C*H$_3$)$_2$), 25.5 (CH(*C*H$_3$)$_2$), 27.6 (*C*H(CH$_3$)$_2$), 28.6 (*C*H(CH$_3$)$_2$), 51.5 (O*Me*), 97.6 (*γ-C*), 123.8 (Ar), 124.6 (Ar), 127.1 (Ar), 140.7 (Ar), 143.2 (Ar), 145.2 (Ar), 169.5 (*C*N). Anal. Calcd for $C_{31}H_{47}AlN_2O$: C, 75.88; H, 9.65; N, 5.71. Found: C, 73.67; H, 9.38; N, 5.54.

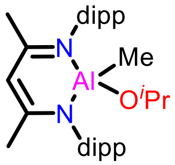

Synthesis of **6c**: Inside the glove box, *iso*-propanol (7.2 µL, 0.09 mmol) was added dropwise to a solution of **1** (50 mg, 0.08 mmol) in toluene (3 mL) at -30 °C. After addition, the reaction was stirred for 3 h at room temperature. The reaction mixture was passed through Celite, and the solvents were removed under reduced pressure to yield a red solid. The red solid was dissolved in toluene (0.5 mL) and pentane (0.5 mL), and the solution was kept at -30 °C overnight. Red solids (Fp$_2$ formed from the remaining FpH) were separated from the colorless solution. Finally, evaporation to dryness yielded **6c** as a colorless solid. Yield: (39 mg, 96%): ^{1}H NMR (C_6D_6, 500 MHz) δ: 1H NMR (500 MHz, C6D6) δ -0.44 (s, 3H, AlCH$_3$). 0.78 (d, *J* = 6.0 Hz, 6H, OCH(CH$_3$)$_2$), 1.14 (d, *J* = 6.9, 12H, CH(CH$_3$)$_2$), 1.32 (d, *J* = 6.7 Hz, 6H, CH(CH$_3$)$_2$), 1.48 (d, *J* = 6.8 Hz, 6H, CH(CH$_3$)$_2$), 1.54 (s, 6H, CH$_3$), 3.17-3.32 (m, 2H, CH(CH$_3$)$_2$), 3.51-3.70 (m, 2H, CH(CH$_3$)$_2$), 3.90 (sept, *J* = 6.0 Hz, 1H, OCH(CH$_3$)$_2$), 4.83 (S, 1H, γ-CH), 6.96 – 7.23 (m, 6H, Ar). ^{13}C{^{1}H} NMR (C_6D_6, 126 MHz): δ 13C NMR (126 MHz, CDCl3) δ -10.2 (AlCH$_3$), 23.2 (CH(CH$_3$)$_2$), 24.4 (CH(CH$_3$)$_2$), 24.6 (CH(CH$_3$)$_2$), 24.8 (CH(CH$_3$)$_2$), 25.7 (CH(CH$_3$)$_2$), 27.4 (CH(CH$_3$)$_2$), 27.6 (CH(CH$_3$)$_2$), 28.4 (CH(CH$_3$)$_2$), 63.4 (OCH), 96.8 (γ-C), 124.1 (Ar), 126.9 (Ar), 140.5 (Ar), 144.1 (Ar), 144.8 (Ar), 169.4 (CN). Anal. Calcd for $C_{33}H_{51}AlN_2O$: C, 76.41; H, 9.91; N, 5.40. Found: C, 75.85; H, 9.76; N, 5.26.

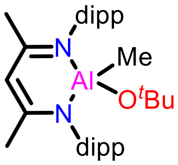

Synthesis of 6d: Inside the glove box, *tert*-butanol (9 µL, 0.09 mmol) was added dropwise to a solution of **1** (50 mg, 0.08 mmol) in toluene (5 mL) at -30 °C. After addition, the reaction mixture was stirred for 3 h at room temperature, and solvent was removed under reduced pressure to get an oily residue. The oily residue was dissolved in pentane (2 mL) and filtered through Celite. Toluene (0.2 mL) was added to the filtrate, and the solution was kept at -30 °C overnight. Red solids (Fp$_2$ formed from the remaining FpH) were separated from the colorless solution. Finally, evaporation to dryness yielded **6d** as colorless solid. Yield: (38 mg, 91%). ^{1}H NMR (C_6D_6, 500 MHz): δ -0.20 (s, 3H, AlC*H*$_3$), 0.92 (s, 9H, C(C*H*$_3$)$_3$), 1.15 (d, 6H, *J* = 7 Hz, CH(C*H*$_3$)$_2$), 1.23 (d, 6H, *J* = 7 Hz, CH(C*H*$_3$)$_2$), 1.42 (d, 6H, *J* = 7 Hz, CH(C*H*$_3$)$_2$), 1.55 (d, 6H, *J* = 7 Hz, CH(C*H*$_3$)$_2$), 1.60 (s, 6H, C*H*$_3$), 3.33-3.40 (m, 2H, C*H*(CH$_3$)$_2$), 3.62-3.68 (m, 2H, C*H*(CH$_3$)$_2$), 4.86 (s, 1H, *γ-CH*), 7.16-7.23 (m, 5H, Ar). ^{13}C{^{1}H}NMR (C_6D_6, 125 MHz): δ -7.9 (AlCH$_3$), 23.2 (*C*H$_3$), 24.0 (CH(*C*H$_3$)$_2$), 24.5 (CH(*C*H$_3$)$_2$), 24.7 (CH(*C*H$_3$)$_2$), 26.1 (CH(*C*H$_3$)$_2$), 27.3 (*C*H(CH$_3$)$_2$), 28.5 (*C*H(CH$_3$)$_2$), 33.4 (C(*C*H$_3$)$_3$), 67.3 (*C*(CH$_3$)$_3$), 96.4 (*γ-C*), 123.8 (Ar), 124.2 (Ar), 126.7 (Ar), 140.8 (Ar), 144.2 (Ar), 144.4 (Ar), 169.5 (*C*N). Anal. Calcd for $C_{34}H_{53}AlN_2O$: C, 76.65; H, 10.03; N, 5.26. Found: C, 76.94; H, 10.32; N, 5.23.

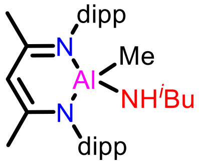

Synthesis of 6e: Inside the glove box, *iso*-butyl amine (4 µL, 0.04 mmol) was added dropwise to a solution of **1** (20 mg, 0.03 mmol) in toluene (2 mL) at -30 °C. After addition, the reaction mixture was stirred for 3 h at room temperature, and solvent was removed under reduced pressure to get an oily residue. The oily residue was dissolved in pentane (2 mL) and filtered through Celite. Compound **6e** was then obtained by evaporating the solution to dryness. Yield: 16 mg (quantitative). ^{1}H NMR (C_6D_6, 500 MHz): δ -0.74 (s, 3H, AlCH_3), 0.17 (t, 1H, J = 7 Hz, AlNH), 0.89 (d, 6H, J = 7 Hz, CH(CH_3)$_2$), 1.13 (d, 6H, J = 7 Hz, CH(CH_3)$_2$), 1.26 (d, 6H, J = 7 Hz, CH(CH_3)$_2$), 1.33 (d, 6H, J = 7 Hz, CH(CH_3)$_2$), 1.39 (d, 6H, J = 7 Hz, CH(CH_3)$_2$), 1.51-1.59 (m, 1H, CH(CH$_3$)$_2$), 1.57 (s, 6H, CH_3), 2.70 (t, 2H, J = 7 Hz, CH_2), 3.25-3.31 (m, 2H, CH(CH$_3$)$_2$), 3.69-3.74 (m, 2H, CH(CH$_3$)$_2$), 4.91 (s, 1H, γ-CH), 7.11-7.18 (m, 5H, Ar). ^{13}C{^{1}H}NMR (C_6D_6, 100 MHz): δ -13.3 (AlCH$_3$), 20.6 (CH(CH$_3$)$_2$), 23.2 (CH$_3$), 24.4 (CH(CH$_3$)$_2$), 24.5 (CH(CH$_3$)$_2$), 25.0 (CH(CH$_3$)$_2$), 25.7 (CH(CH$_3$)$_2$), 27.6 (CH(CH$_3$)$_2$), 28.3 (CH(CH$_3$)$_2$), 32.9 (CH(CH$_3$)$_2$), 54.2 (CH$_2$), 97.6 (γ-C), 124.1 (Ar), 124.4 (Ar), 126.8 (Ar), 141.3 (Ar), 143.7 (Ar), 144.8 (Ar), 169.1 (CN).

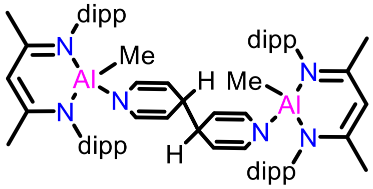

Synthesis of 2: Inside the glove box, pyridine (19 µL, 0.24 mmol) was added dropwise to a solution of **1** (150 mg, 0.24 mmol) in toluene (15 mL) at -30 °C. After addition, the reaction mixture was stirred for 3 h at room temperature, and solvent was removed under reduced pressure to get a red oily residue. The oily residue was washed with pentane (4 mL), resulting in a yellow-red solid. The solid was dissolved in a 1:1 mixture of toluene and pentane (total volume: 5 mL) and kept at -30 °C overnight. This process was repeated twice, resulting in yellow colored compound **2**. Yellow crystals suitable for XRD analysis were grown from a mixture of toluene and pentane at −40 °C. Yield: (58 mg, 46%). ^{1}H NMR (C_6D_6, 500 MHz): δ -0.87 (s, 3H, AlCH_3), 1.04 (d, 6H, J = 7 Hz, CH(CH_3)$_2$), 1.29 (d, 6H, J = 7 Hz, CH(CH_3)$_2$), 1.39 (d, 6H, J = 7 Hz, CH(CH_3)$_2$), 1.54 (s, 6H, CH_3), 1.67 (bs, 6H, CH(CH_3)$_2$), 3.16-3.22 (m, 2H, CH(CH$_3$)$_2$), 3.62-3.68 (m, 2H, CH(CH$_3$)$_2$), 3.80 (s, 2H, Py), 4.89 (s, 3H, γ-CH & Py), 6.27 (bs, 2H, Py), 7.07 (d, 2H, J = 8 Hz, Ar), 7.12-7.15 (m, 1H, Ar), 7.20 (d, 2H, J = 8 Hz, Ar). ^{13}C{^{1}H}NMR (C_6D_6, 100 MHz): δ -15.9 (AlCH$_3$) , 23.2 (CH$_3$), 24.1 (CH(CH$_3$)$_2$), 24.6 (CH(CH$_3$)$_2$), 24.7 (CH(CH$_3$)$_2$), 26.4 (CH(CH$_3$)$_2$), 28.7 (CH(CH$_3$)$_2$), 43.9 (Py), 98.2 (γ-C), 101.5 (Py), 123.7 (Ar), 125.0 (Ar), 127.2 (Ar), 132.9 (Py), 140.3 (Ar), 143.0 (Ar), 146.1 (Ar), 170.2 (CN). Anal. Calcd for $C_{70}H_{98}Al_2N_6$: C, 78.03; H, 9.17; N, 7.80. Found: C, 75.73; H, 9.07; N, 7.50.

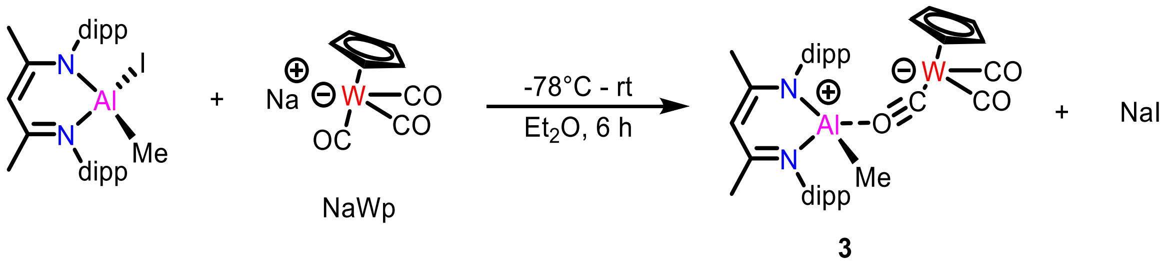

Supplementary Figure 1. Synthesis of **3**

Synthesis of 3: Inside the glove box, NaWp (50 mg, 0.14 mmol) was suspended in diethyl ether (10 mL) and subsequently added dropwise to a solution of $L^{dipp}Al(Me)(I)$ (100 mg, 0.13 mmol) in diethyl ether (5 mL) at -78 °C. After addition, the reaction was stirred for 6 h at room temperature. The reaction mixture was filtered through Celite, and the solvents were removed under reduced pressure to yield a pale-yellow solid. Finally, the yellow solid was dissolved in toluene (1 mL) and pentane (15 mL) and left overnight for precipitation. The supernatant was decanted, and the solids were dried *in vacuo* to yield **3** as a pale-yellow solid. Yellow crystal suitable for XRD analysis were grown from the mixture of toluene and pentane. Yield: (88 mg, 71 %). ^{1}H NMR (C_6D_6, 500 MHz): δ -0.95 (s, 3H, AlCH3), 0.99 (d, *J* = 6.9 Hz, 6H, $CH(CH_3)_2$), 1.20 (d, *J* = 7.0 Hz, 6H, $CH(CH_3)_2$), 1.40 (d, *J* = 6.7 Hz, 6H, $CH(CH_3)_2$), 1.54 (d, *J* = 6.7 Hz, 6H, $CH(CH_3)_2$), 1.76 (s, 6H, CH_3), 3.03 (sept, *J* = 6.9 Hz, 2H, $CH(CH_3)_2$), 3.74 (sept, *J* = 6.7 Hz, 2H, $CH(CH_3)_2$), 5.17 (s, 2H, Cp), 5.36 (s, 1H, γ-CH), 7.01-7.06 (s, 2H, Ar), 7.13-7.18 (m, 2H, Ar), 7.19-7.23 (m, 2H, Ar). ^{13}C{^{1}H} NMR (C_6D_6, 126 MHz): δ -16.9 ($AlCH_3$), 23.5($CH(CH_3)_2$), 24($CH(CH_3)_2$), 24.2($CH(CH_3)_2$), 24.3 ($CH(CH_3)_2$), 26 ($CH(CH_3)_2$), 27.8 ($CH(CH_3)_2$), 28.8 ($CH(CH_3)_2$), 87.9 (Cp), 100.2 (γ-C), 123.9 (Ar), 125.1 (Ar), 138.8 (Ar), 142.8 (Ar), 145.8 (Ar), 171.8 (CN), 221.8 (CO), 238.14 (CO). IR (solid, cm^{-1}): 1936 (νCO), 1846 (νCO). Anal. Calcd for $C_{38}H_{49}AlN_2O_3W$: C, 57.58; H, 6.23; N, 3.53. Found: C, 57.74; H, 6.33; N, 3.55.

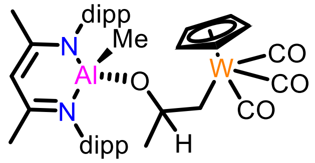

Synthesis of 4: Inside the glove box, (±)-propylene oxide (11 µL, 0.15 mmol) was added dropwise to a solution of **3** (100 mg, 0.14) in toluene (5 mL) at -78 °C. After addition, the reaction was stirred overnight at room temperature. The reaction mixture was passed through Celite, and the solvent was removed under reduced pressure to yield a pale-yellow solid. Finally, the yellow solid was washed with pentane and dried in vacuo to yield **4** in pure form. Yield: (80 mg, 68 %). ^{1}H NMR (C_6D_6, 500 MHz): δ-0.42 (s, 3H, $AlCH_3$), 0.96 (d, *J* = 6.1 Hz, 3H, CH_3), 1.18 – 1.11 (m, 12H, $CH(CH_3)_2$), 1.35 – 1.28 (m, 6H, $CH(CH_3)_2$), 1.44 – 1.38 (m, 2H, CH_2W), 1.57 – 1.48 (m, 12H, CH_3, $CH(CH_3)_2$), 3.25 (sept, *J* = 6.8 Hz, 2H), 3.64 (sept, *J* = 6.8 Hz, 2H), 4.01 (m, 1H), 4.59 (s, 5H, Cp), 4.86 (s, 1H, γ-CH), 7.23 – 7.02 (m, 6H, Ar). ^{13}C{^{1}H} NMR (C_6D_6, 126 MHz): δ -10.3 ($AlCH_3$), 1.5 (WCH_2), 14 ($OCHCH_3$), 22.4 (CH_3), 23.3 ($CH(CH_3)_2$), 24.4 ($CH(CH_3)_2$), 24.6 ($CH(CH_3)_2$), 25.7 ($CH(CH_3)_2$), 25.8 ($CH(CH_3)_2$), 27.6 ($CH(CH_3)_2$), 28.3 ($CH(CH_3)_2$), 28.4 ($CH(CH_3)_2$), 34.1 ($CH(CH_3)_2$), 75 (OCH), 90.8 (Cp), 97 (γ-C), 124.2 (Ar), 124.3 (Ar), 126.9 (Ar), 140.8 (Ar), 144.2 (Ar), 144.3 (Ar) , 144.8 (Ar), 169.4 (CN), 218.2 (CO), 218.5 (CO), 230.10 (CO). IR (solid, cm–1

): 2004 (νCO), 1917 (νCO), 1889 (νCO). Anal. Calcd for $C_{41}H_{55}AlN_2O_4W$: C, 57.89; H, 6.52; N, 3.29. Found: C, 57.82; H, 6.51; N, 3.28.

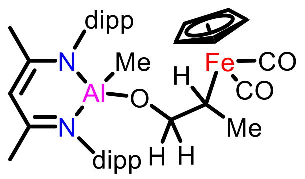

Synthesis of 5: Inside the glove box, (±)-propylene oxide (12 µL, 0.17 mmol) was added dropwise to a solution of **1** (100 mg, 0.16) in toluene (5 mL) at -78 °C. After addition, the reaction was stirred overnight at room temperature. The reaction mixture was passed through Celite, and the solvent was removed under reduced pressure to yield a yellow solid. Finally, the yellow solid was washed with pentane and dried *in vacuo* to yield **5** in pure form. Yellow crystal suitable for XRD analysis were grown from the mixture of toluene and pentane. Yield: (92 mg, 82 %). ^{1}H NMR (C_6D_6, 500 MHz): -0.40 (s, 3H, $AlCH_3$), 1.02 (d, *J* = 6.0 Hz, 3H, $FeCH(CH_3)$), 1.14 (d, *J* = 6.9 Hz, 12H, $CH(CH_3)_2$), 1.21 – 1.30 (m, 1H,$CH_2CH(Fe)CH_3$), 1.34 (d, *J* = 6.7 Hz, 6H, $CH(CH_3)_2$), 1.52 (d, *J* = 6.7 Hz, 6H, $CH(CH_3)_2$), 1.55 (s, 6H, CH_3), 3.21 – 3.30 (m, 2H, $CH(CH_3)_2$), 3.57 – 3.69 (m, 2H, $CH(CH_3)_2$), 3.69 – 3.78 (m, 2H, OCH_2), 4.03 (s, 5H, Cp), 4.83 (s, 1H, γ-CH), 7.05 – 7.10 (m, 2H, Ar), δ 7.21 – 7.11 (m, 4H, Ar). $^{13}C\{^{1}H\}$ NMR (C_6D_6, 126 MHz): *δ* -9.9 ($AlCH_3$), 14.4 ($FeCH(CH_3)$), 23.3 (CH_3), 24.5 ($CH(CH_3)_2$), 24.6 ($CH(CH_3)_2$), 24.7 ($CH(CH_3)_2$), 24.8 ($CH(CH_3)_2$), 27 ($CH(CH_3)_2$), 27.6 ($CH(CH_3)_2$), 28.4 ($CH(CH_3)_2$), 28.5 ($CH(CH_3)_2$), 74 (CH), 84.9 (Cp), 96.7 (γ-C), 124.1 (Ar), 124.2 (Ar), 126.8 (Ar), 126.9 (Ar), 140.7 (Ar), 140.8 (Ar), 144.2 (Ar), 144.3 (Ar), 144.9 (Ar), 169.3 (CN), 217.8 (CO), 218.3 (CO). IR (solid, cm^{-1}): 1992 (νCO), 1934 (νCO). Anal. Calcd for $C_{40}H_{55}AlFeN_2O_3$: C, 69.16; H, 7.98; N, 3.88. Found: C, 68.02; H, 7.98; N, 3.90.

Other relevant reactivity experiments with 3

Inside the glove box, an amine, alcohol, water, or pyridine (2 equiv.) was added dropwise to a solution of **3** (1 equiv.) in toluene (5 mL) at -78 °C. After addition, each solution was stirred overnight at room temperature. In the cases of *iso*-propyl amine, *iso*-butyl amine, and pyridine, analysis by ^{1}H NMR indicated recovery of only starting material. In the cases of *iso*-propanol, *tert*-butanol, and water, ^{1}H NMR analysis indicated quantitative conversion at room temperature to the expected L^{dipp}Al(Me)(OR) complex with release of WpH.

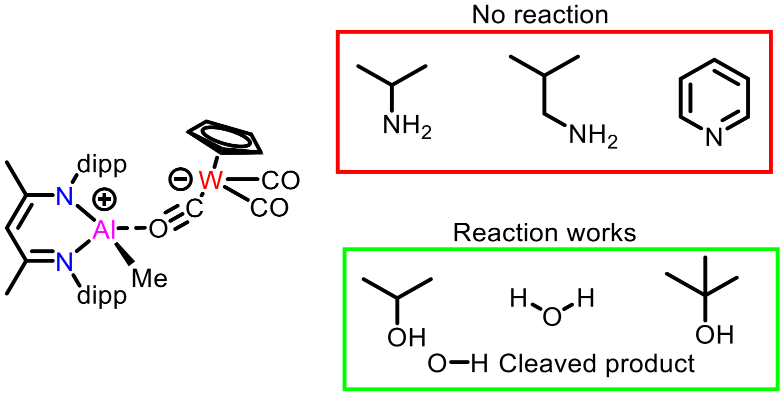

Supplementary Figure 2. Reactivity of Al/Wp with alcohol

2. NMR Spectra

Spectra for compound 6a

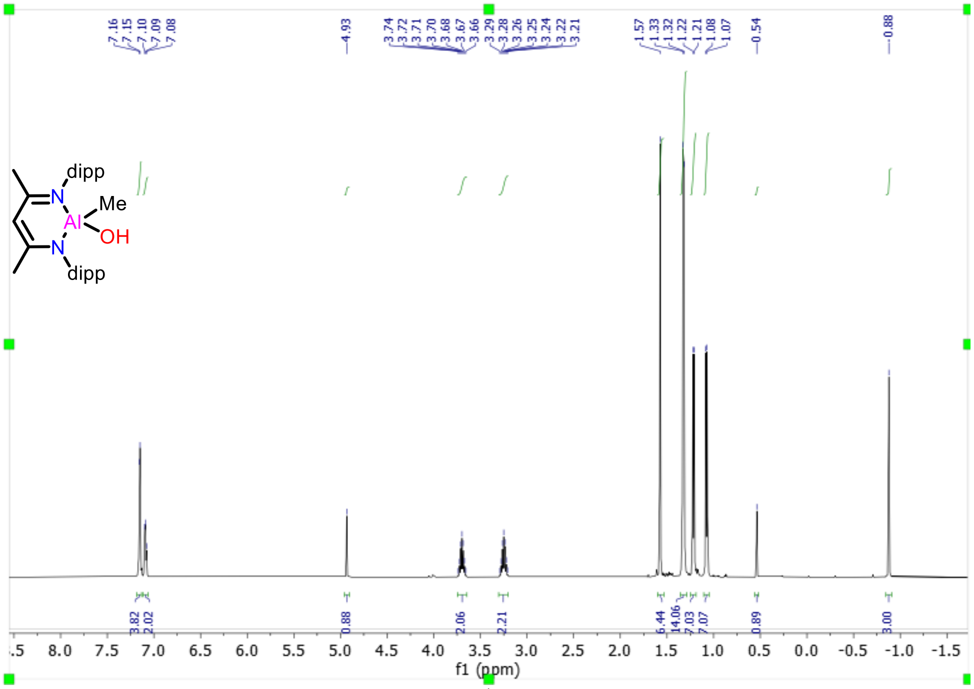

Supplementary Figure 3. ^{1}H NMR at 298K in C_6D_6

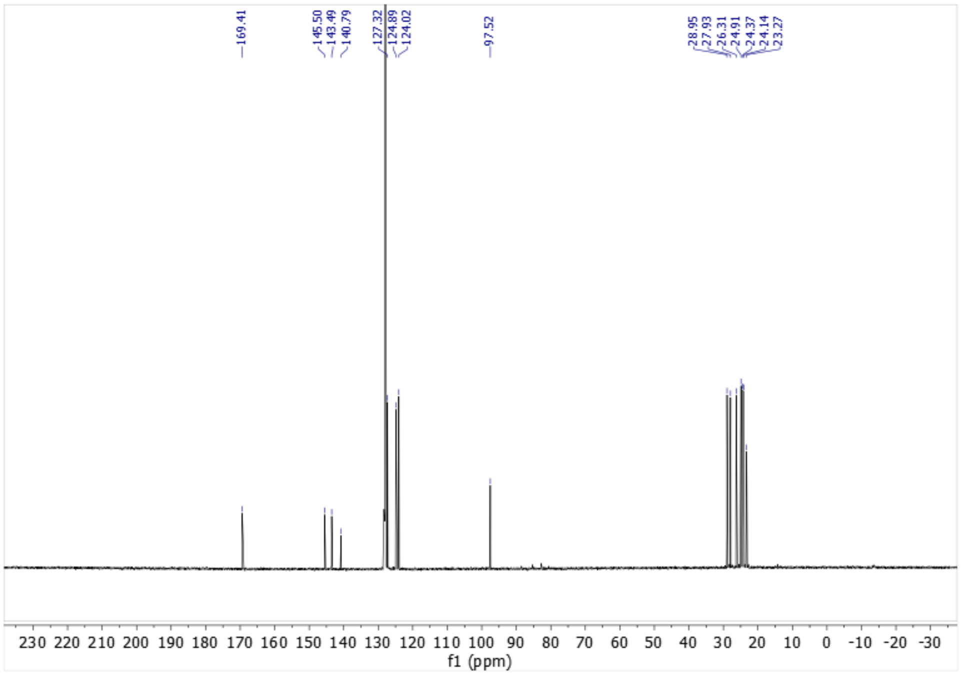

Supplementary Figure 4. $^{13}C\{^{1}H\}$ NMR at 298K in C_6D_6

Spectra for compound 6b

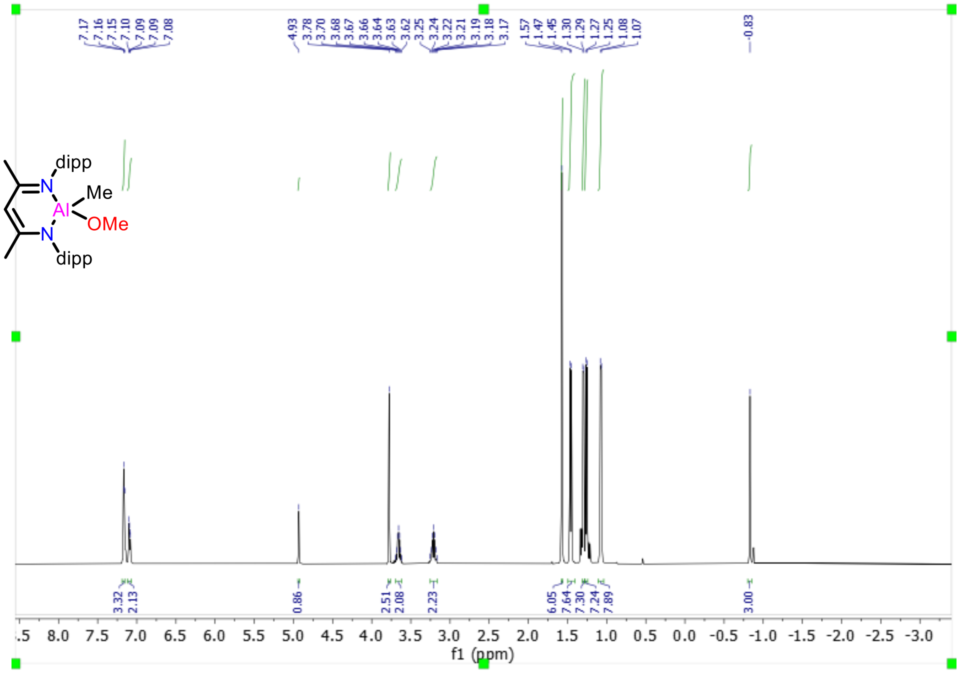

Supplementary Figure 5. ^{1}H NMR at 298K in C_6D_6

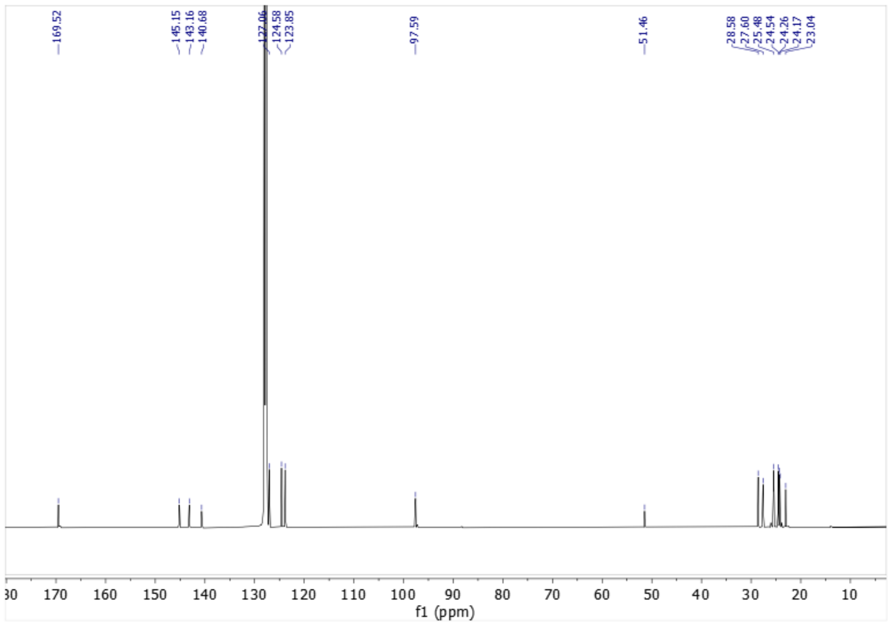

Supplementary Figure 6. $^{13}C\{^{1}H\}$ NMR at 298K in C_6D_6

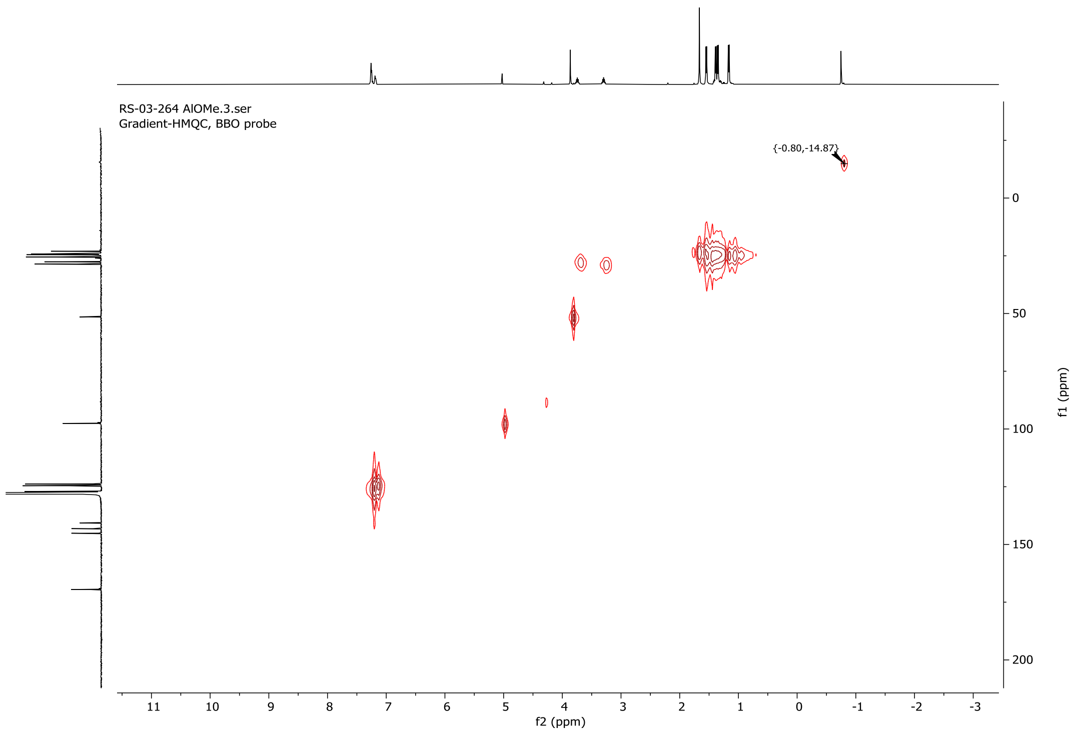

Supplementary Figure 7. ^{1}H- ^{13}C HMQC NMR at 298K in C_6D_6

Spectra for compound 6c

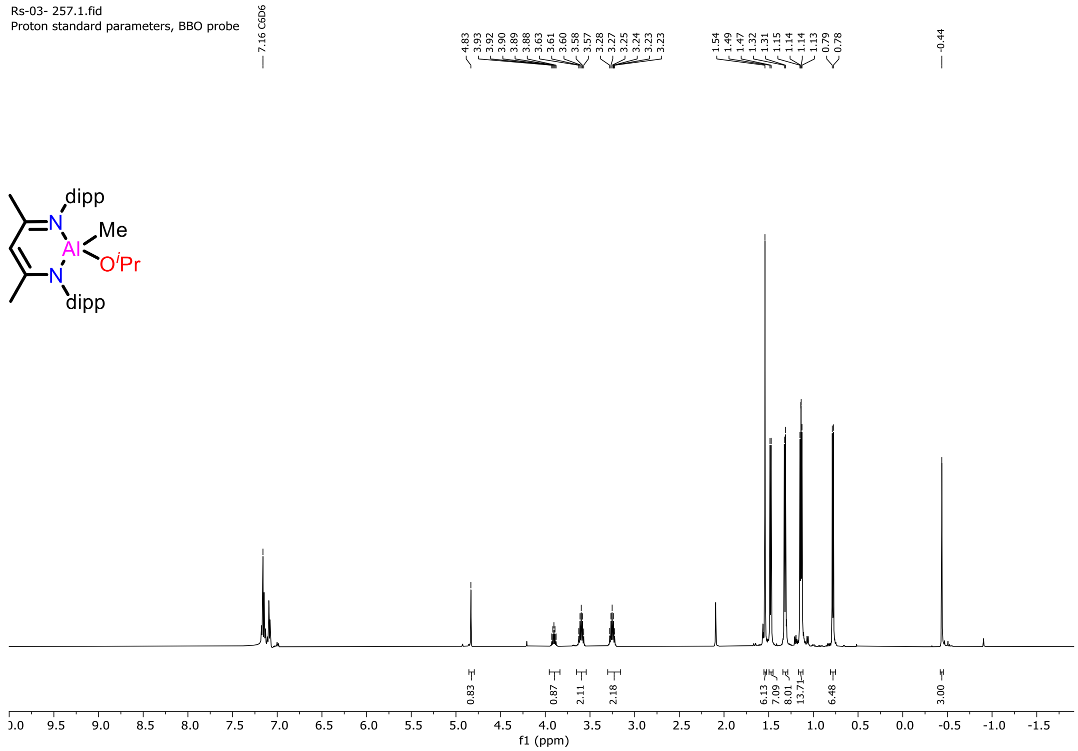

Supplementary Figure 8. ^{1}H NMR at 298K in C_6D_6

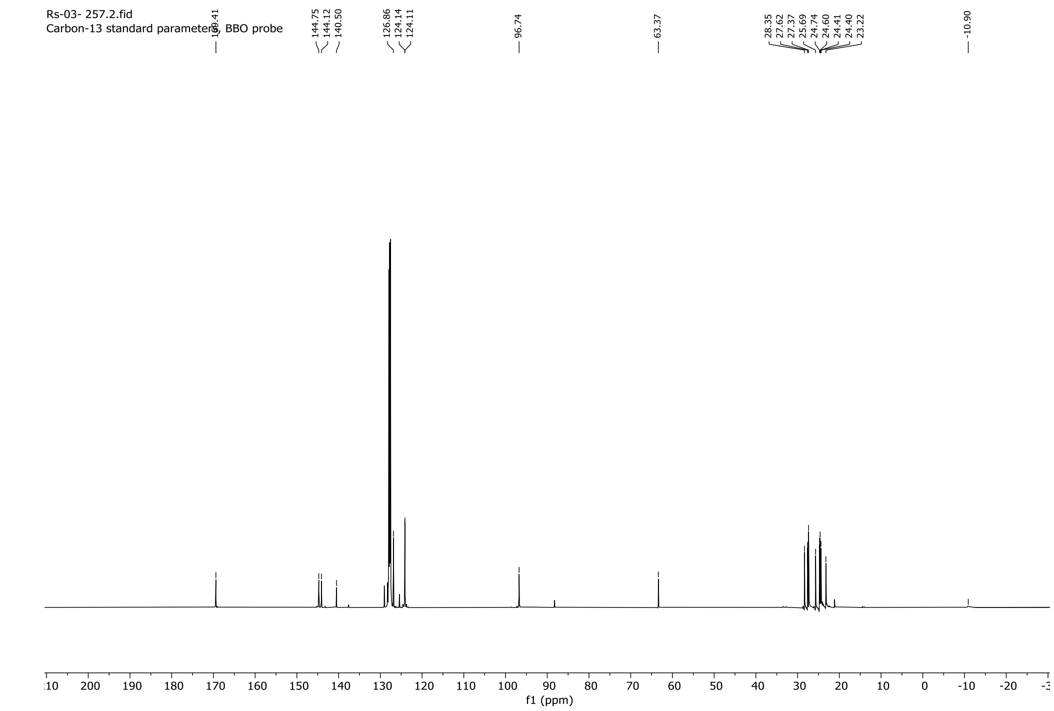

Supplementary Figure 9. $^{13}C\{^{1}H\}$ NMR at 298K in C_6D_6

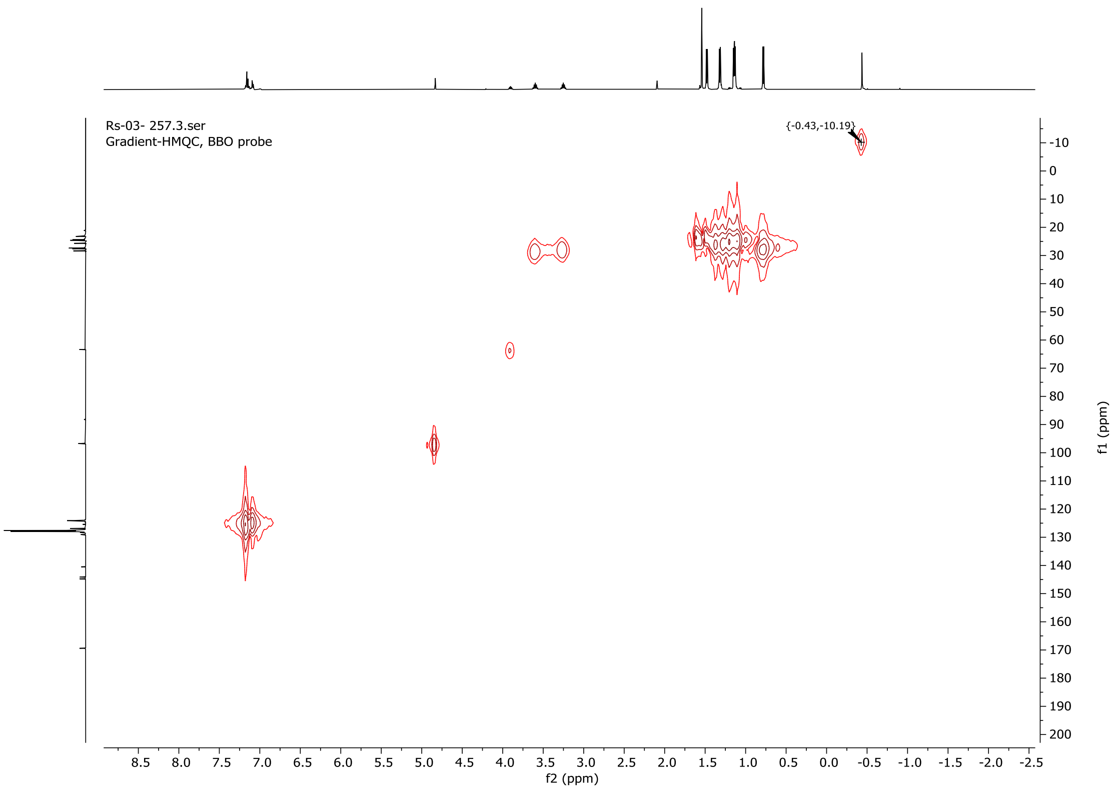

Supplementary Figure 10. ^{1}H- ^{13}C HMQC NMR at 298K in C_6D_6

Spectra for compound 6d

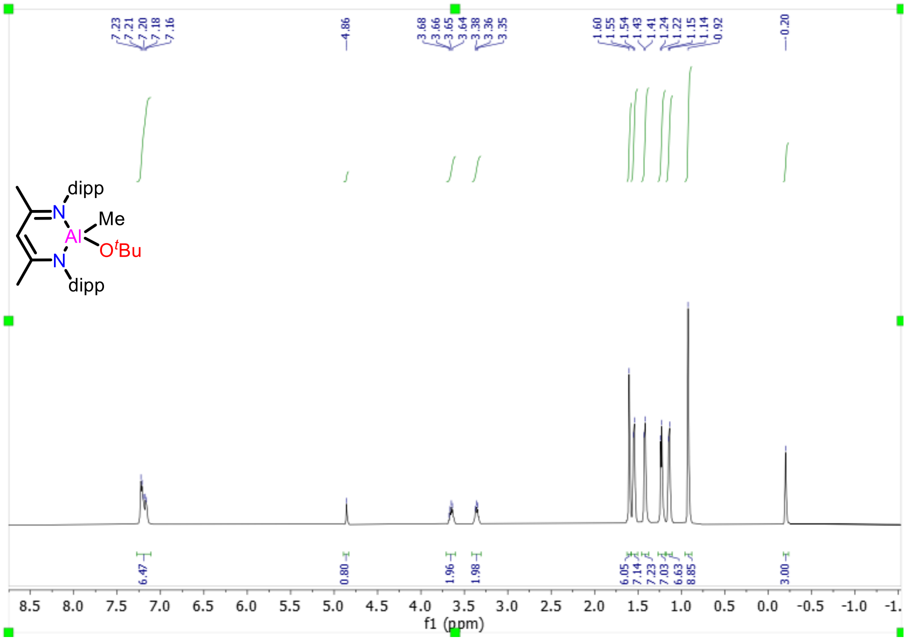

Supplementary Figure 11. ^{1}H NMR at 298K in C_6D_6

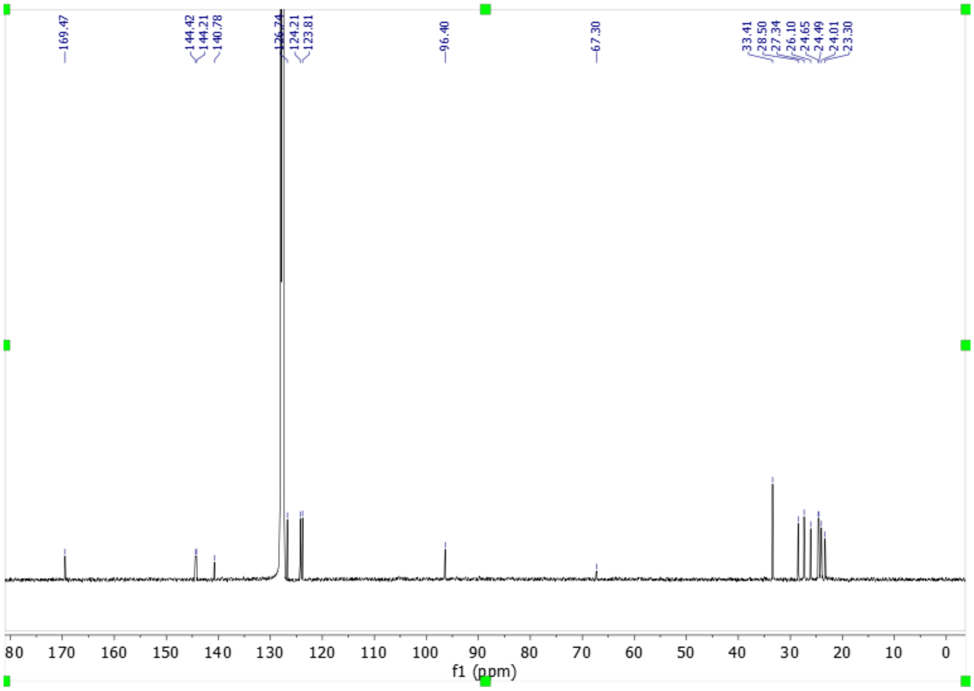

Supplementary Figure 12. $^{13}C\{^{1}H\}$ NMR at 298K in C_6D_6

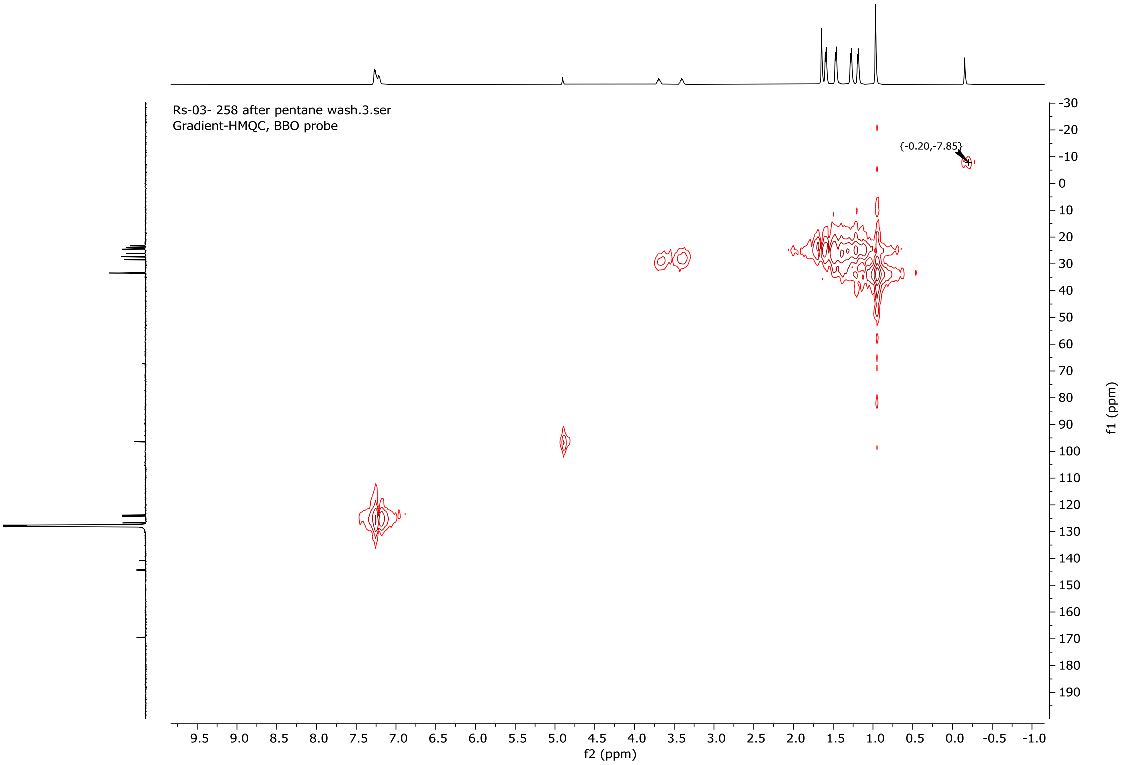

Supplementary Figure 13. ^{1}H- ^{13}C HMQC NMR at 298K in C_6D_6

Spectra for compound 6e

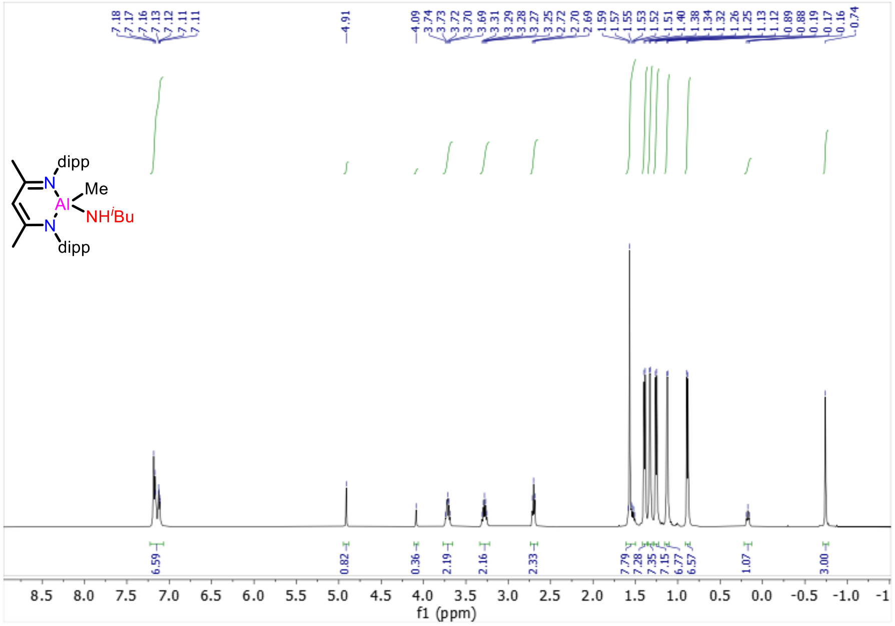

Supplementary Figure 14. ^{1}H NMR at 298K in C_6D_6

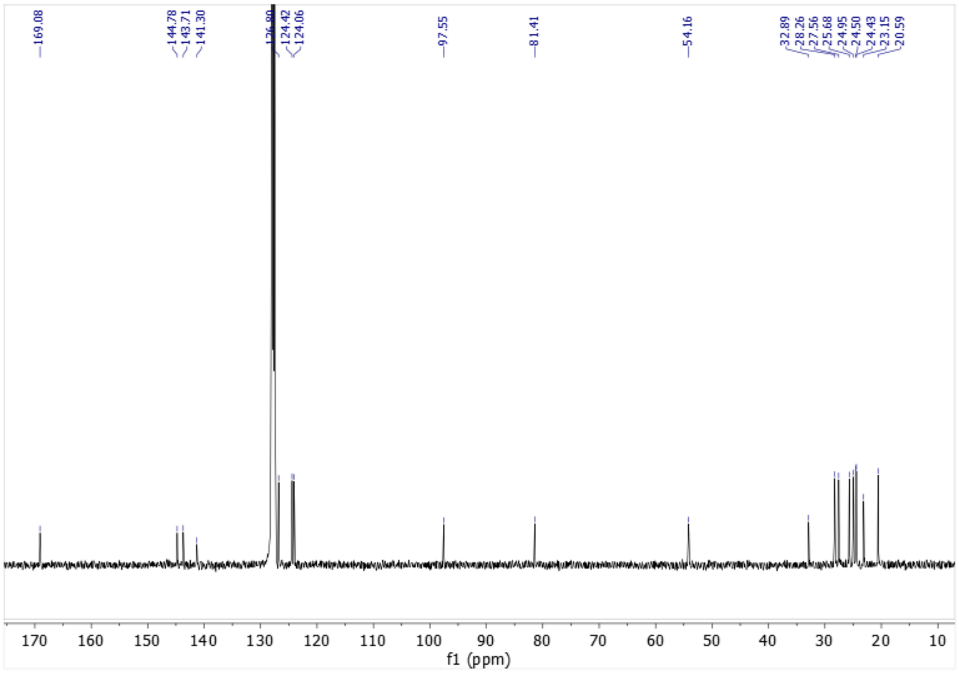

Supplementary Figure 15. $^{13}C\{^{1}H\}$ NMR at 298K in C_6D_6

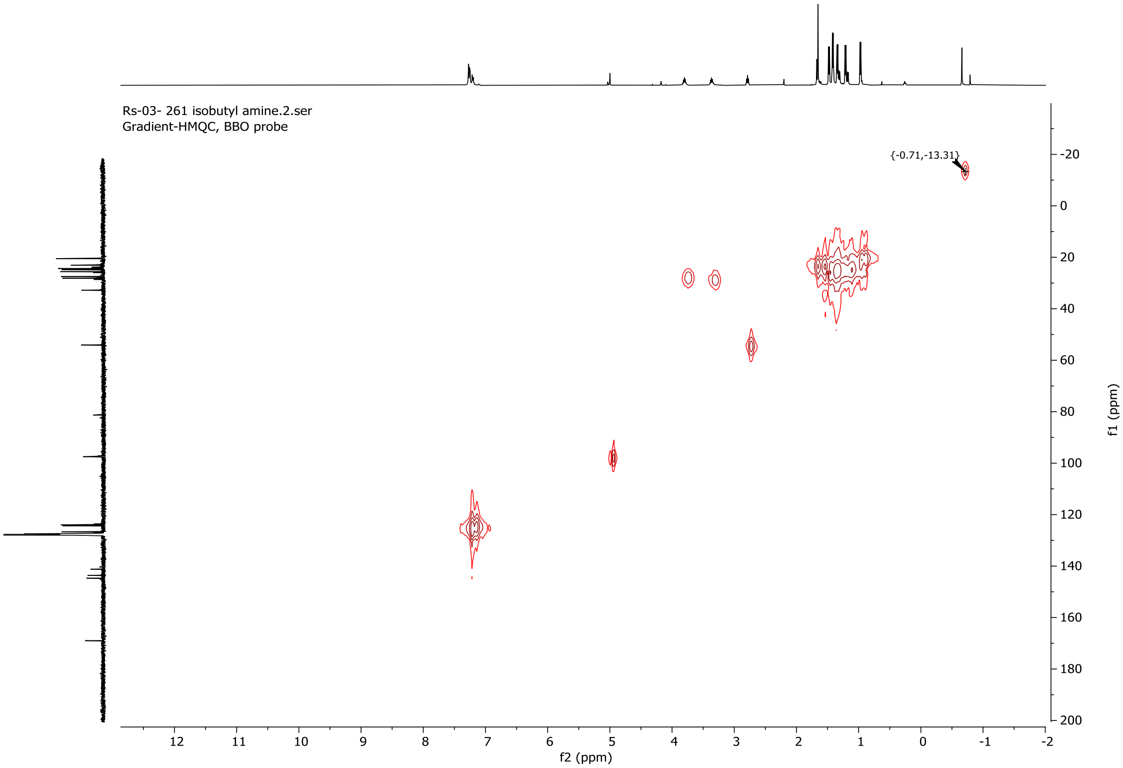

Supplementary Figure 16. ^{1}H- ^{13}C HMQC NMR at 298K in C_6D_6

Spectra for compound 2

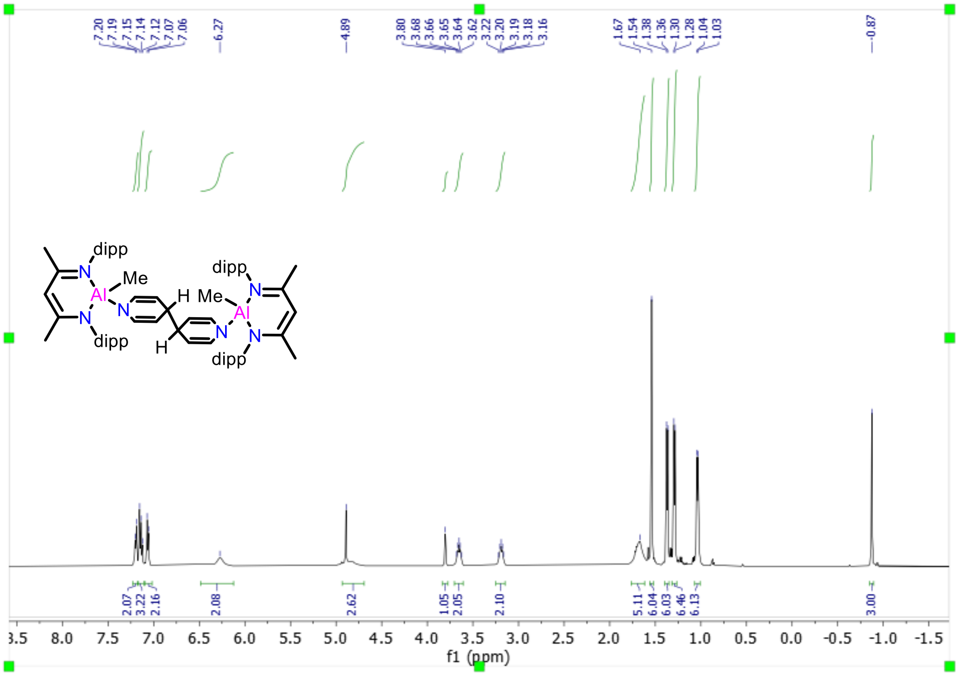

Supplementary Figure 17. ^{1}H NMR at 298K in C_6D_6

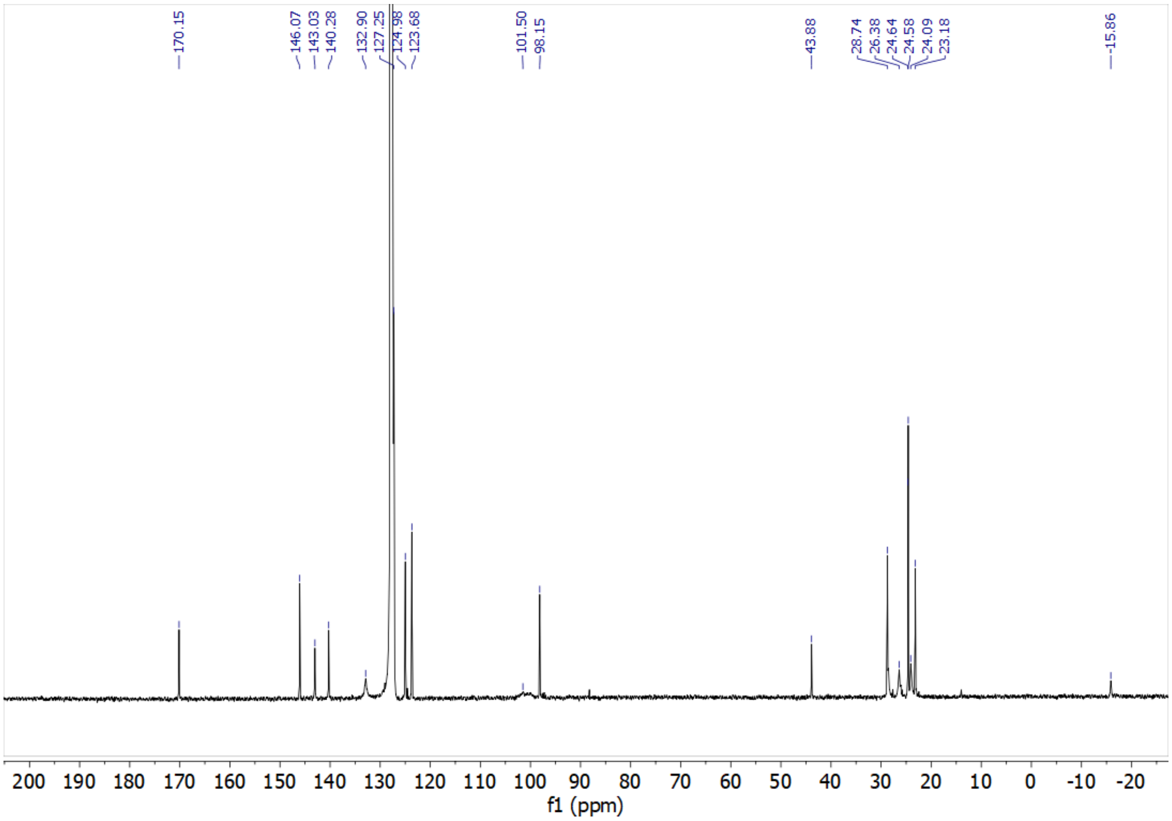

Supplementary Figure 18. $^{13}C\{^{1}H\}$ NMR at 298K in C_6D_6

Spectra for compound 3

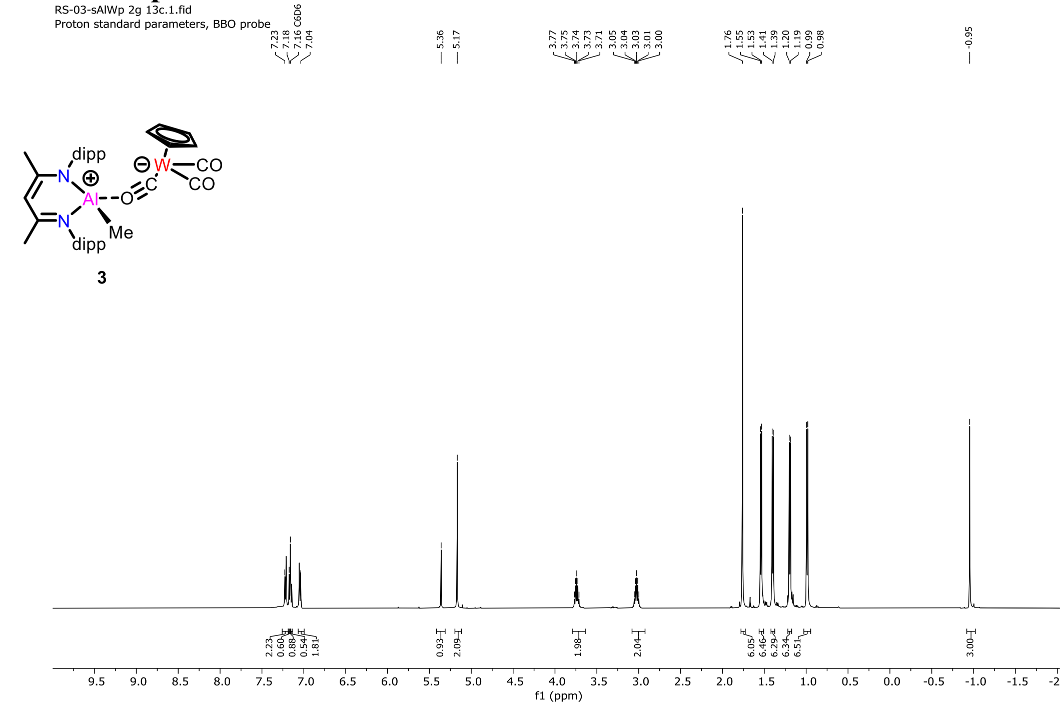

Supplementary Figure 19. ^{1}H NMR at 298K in C_6D_6

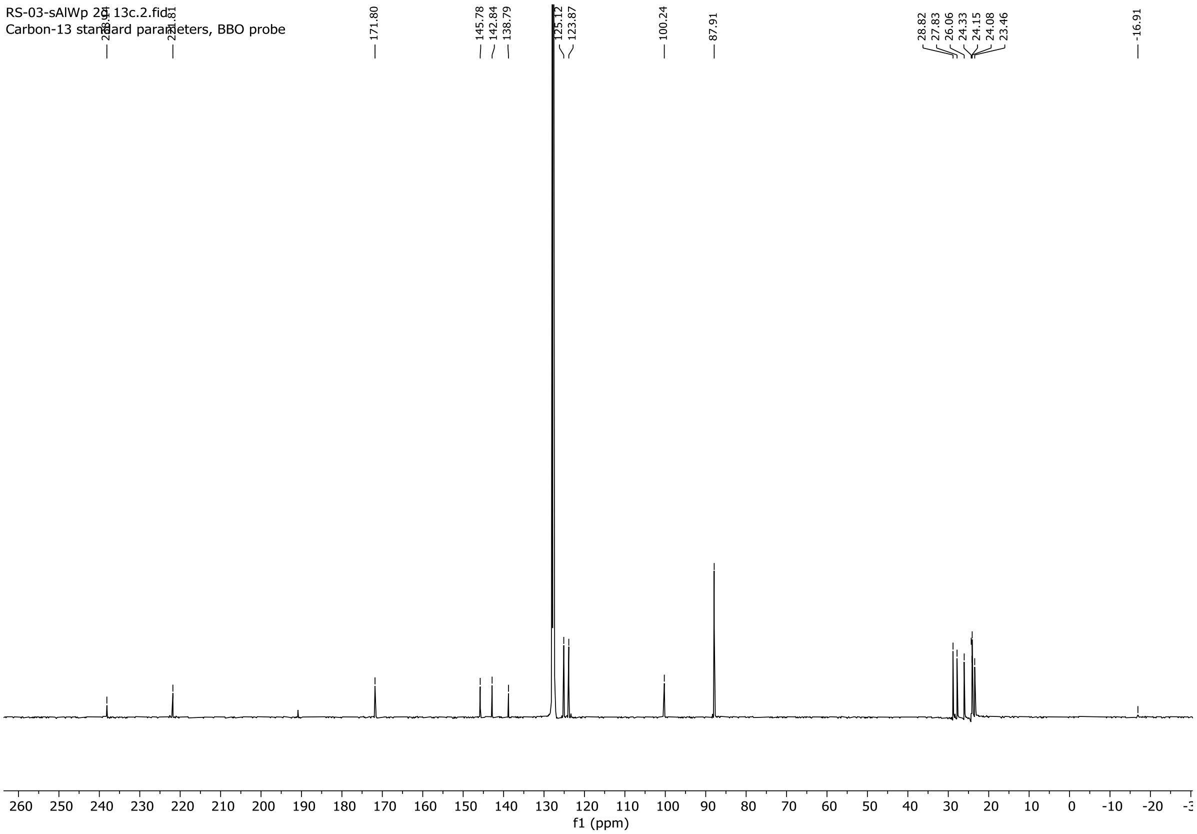

Supplementary Figure 20. $^{13}C\{^{1}H\}$ NMR at 298K in C_6D_6

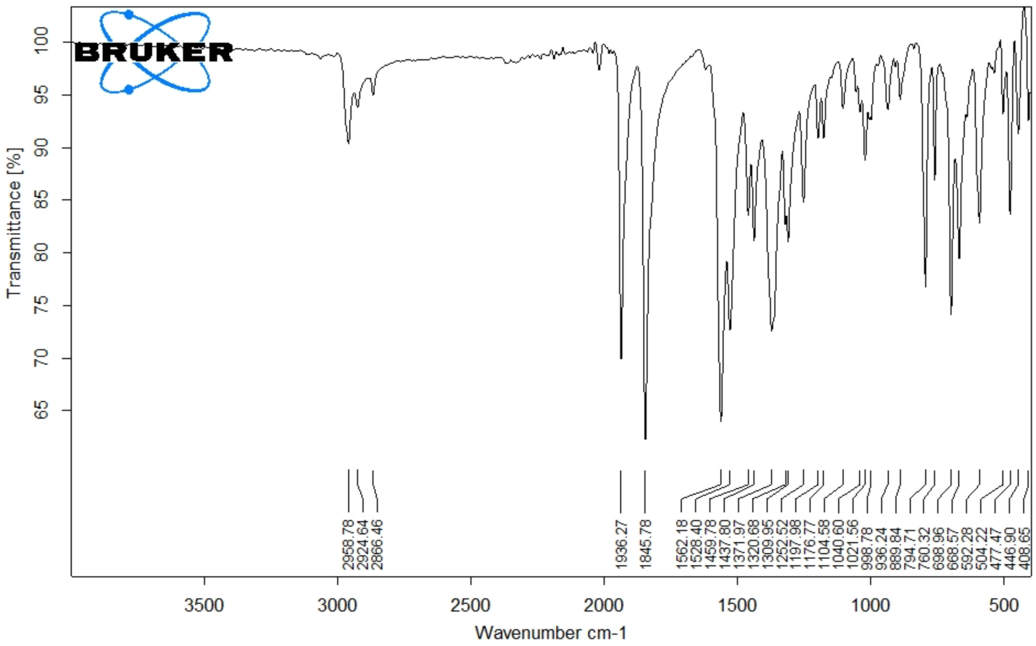

Supplementary Figure 21. Solid state FT-IR of neat 3

Spectra for compound 4

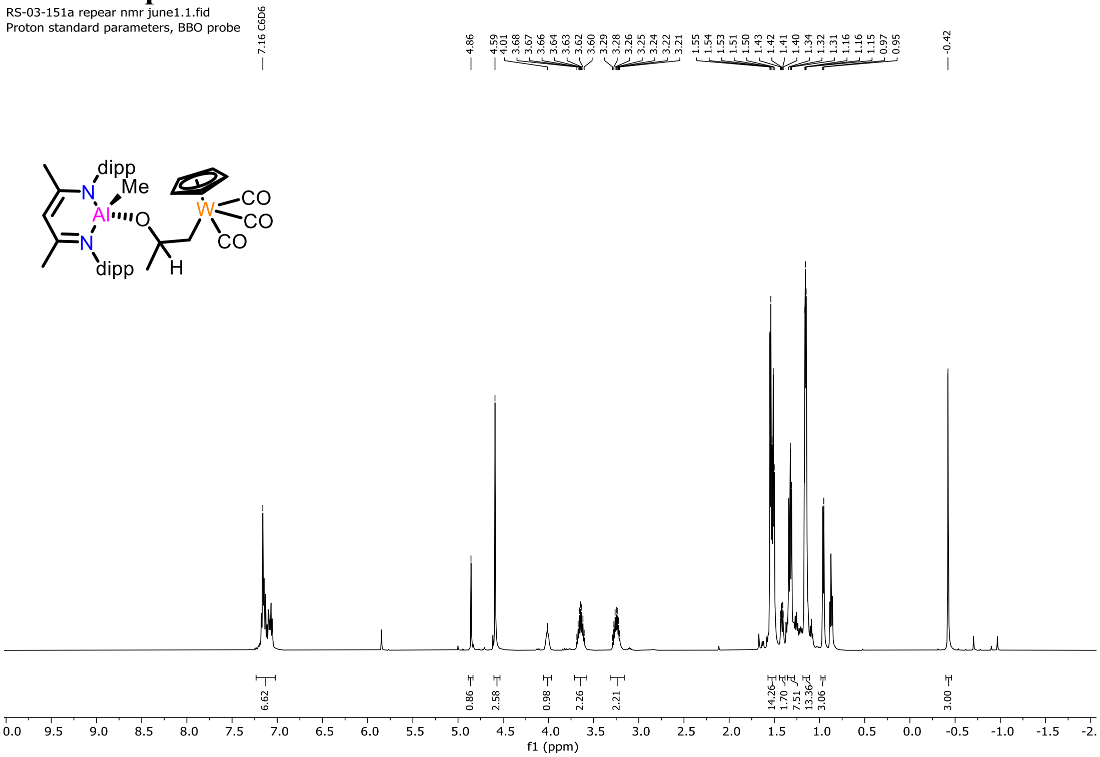

Supplementary Figure 22. ^{1}H NMR at 298K in C_6D_6

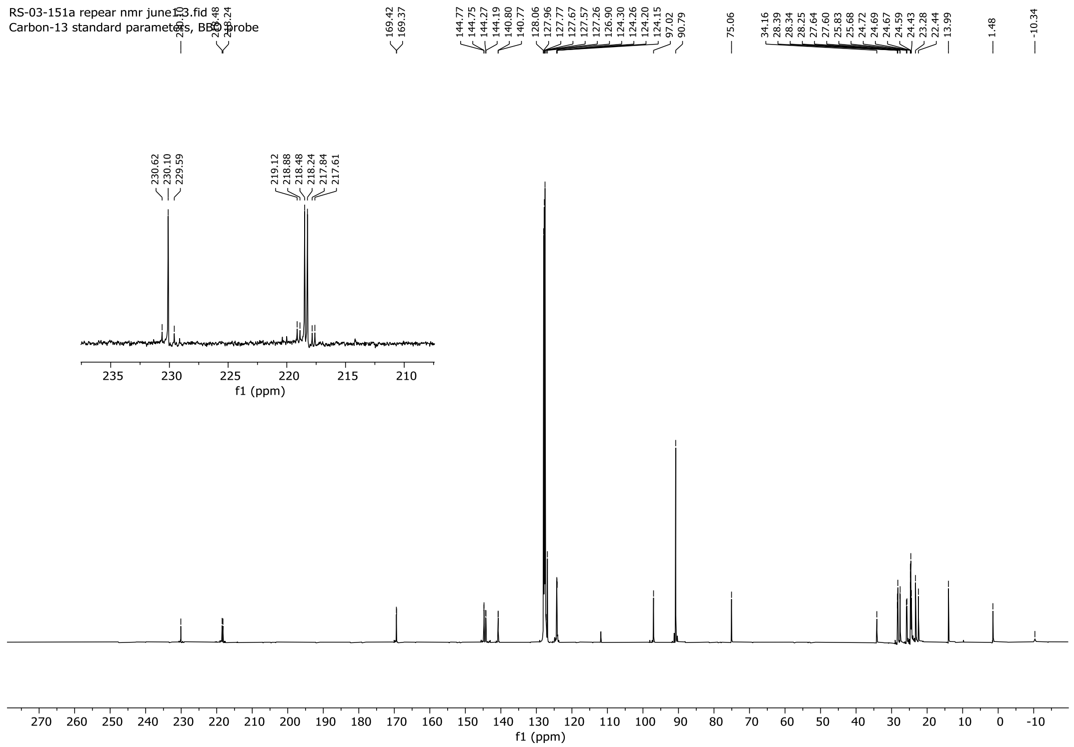

Supplementary Figure 23. $^{13}C\{^{1}H\}$ NMR at 298K in C_6D_6

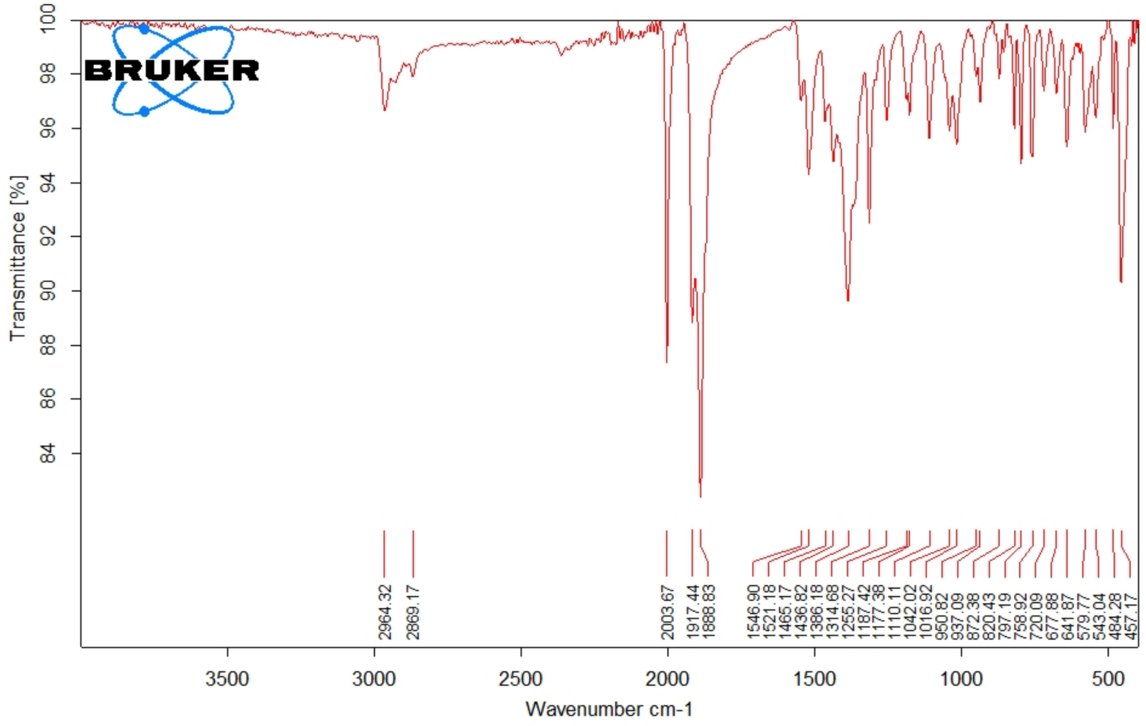

Supplementary Figure 24. Solid state FT-IR of neat 4

Spectra for compound 5

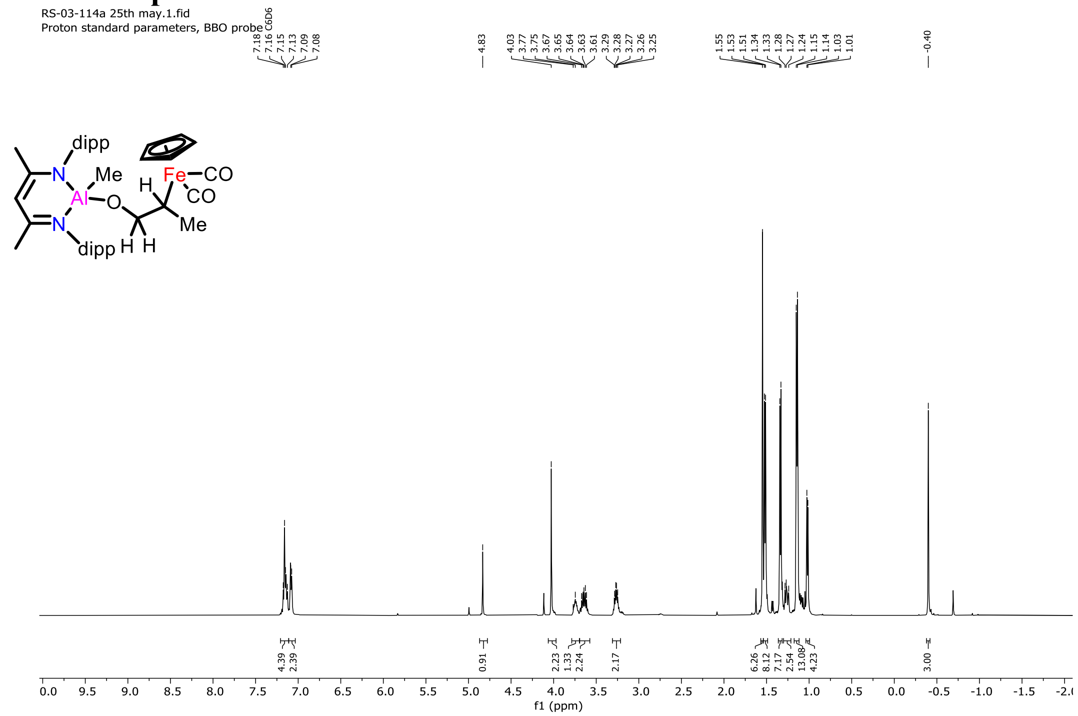

Supplementary Figure 25. ^{1}H NMR at 298K in C_6D_6

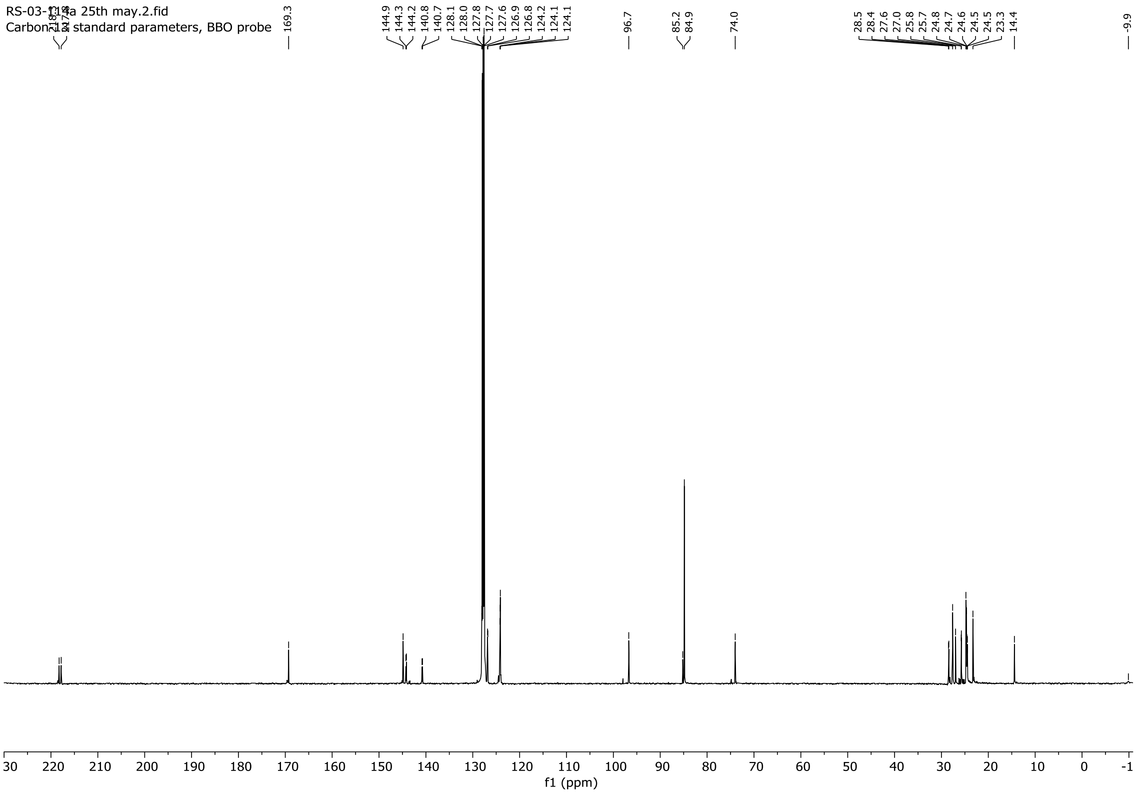

Supplementary Figure 26. $^{13}C\{^{1}H\}$ NMR at 298K in C_6D_6

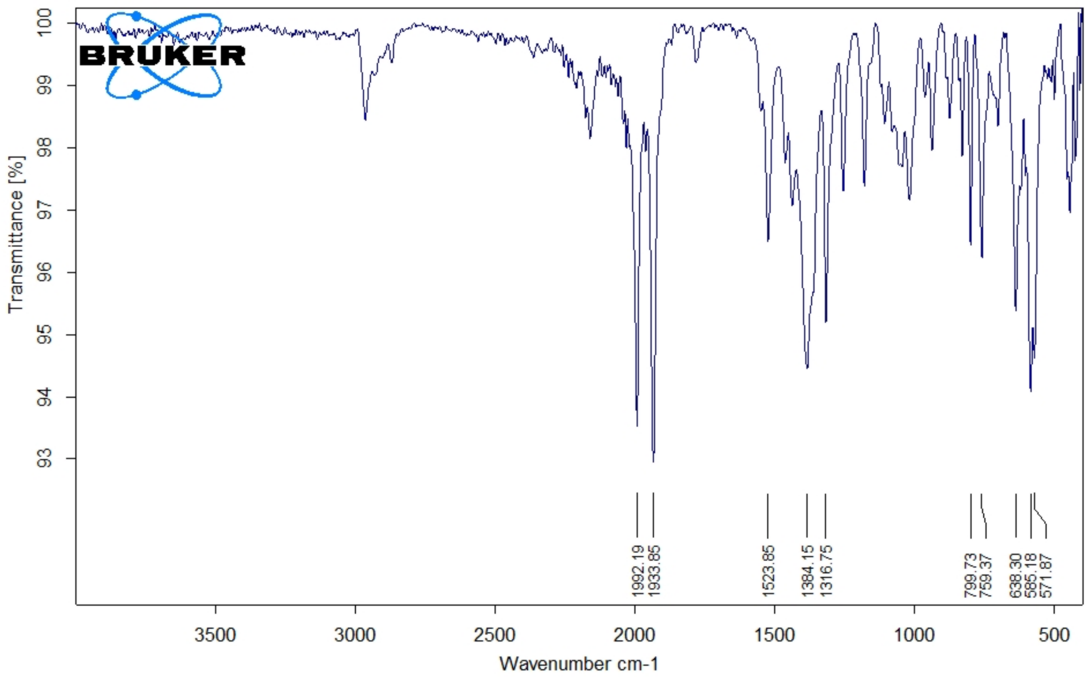

Supplementary Figure 27. Solid state FT-IR of neat 5

Spectra for crude reaction mixture of 6e

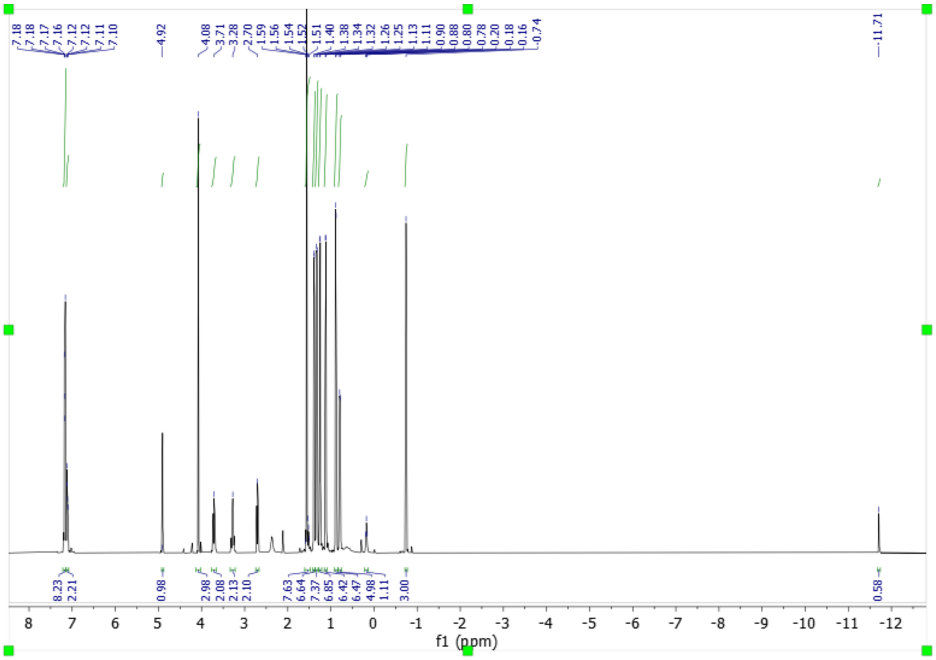

Supplementary Figure 28. ^{1}H NMR at 298K reaction mixture of **6e** in C_6D_6

3. Kinetic Experiments

General Procedure for the Rate Measurements: 2 (10.0 mg, 0.016 mmol) was dissolved in toluene-d_8 (0.4 mL), transferred to a NMR tube, and sealed using a rubber septum and Teflon tape. The NMR tube was then taken out of the glovebox and dipped in liquid nitrogen to freeze the solution. Alcohol or amine (6 equiv.) in toluene-d_8 (0.1 mL) was added by syringe to the frozen sample, and then the NMR tube was immediately transferred to a dry ice/acetone bath and allowed to warm to –78 °C. Finally, the tube was shaken to ensure mixing and then quickly transferred into the NMR probe which was precooled to 283 K.

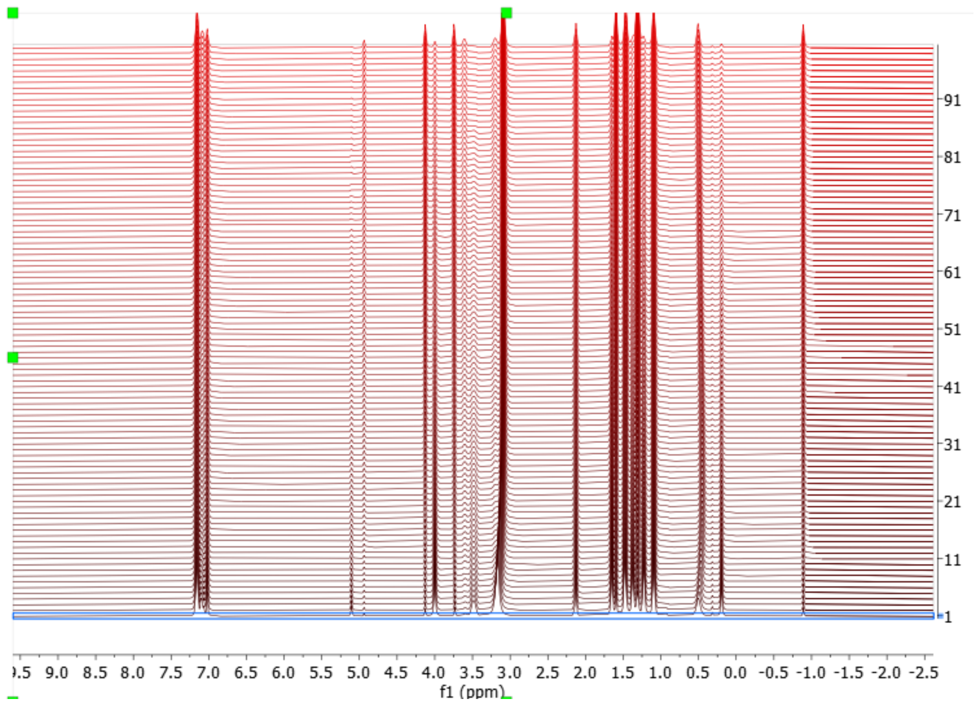

Supplementary Figure 29. Representative stacked ^{1}H NMR spectra from monitoring the reaction of **1** with methanol at 283 K. The spectra were obtained at 3 min intervals.

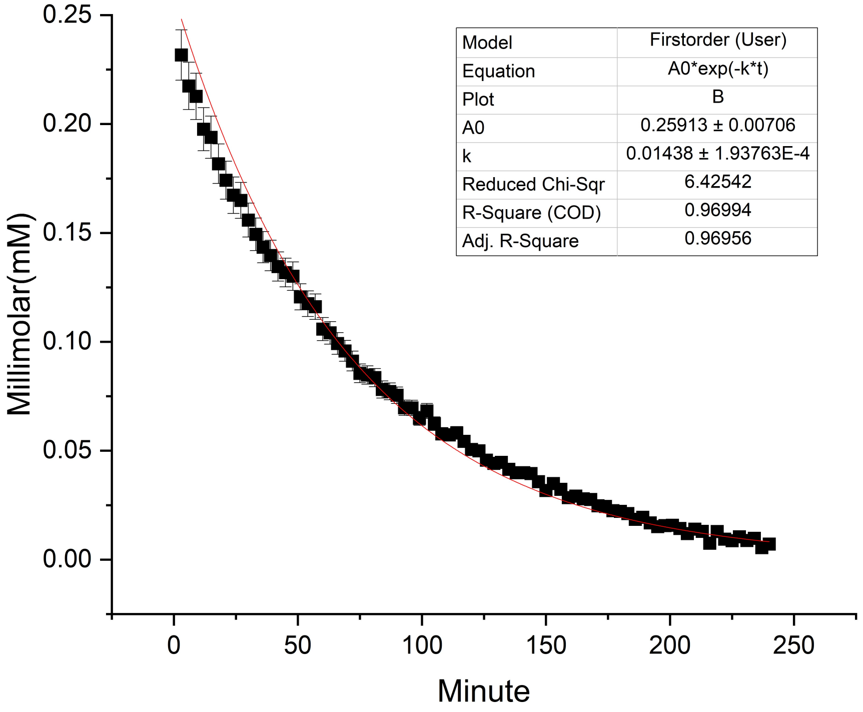

Supplementary Figure 30. Representative plot of starting material concentration vs. time at 283 K for the reaction with MeOH. Pseudo-first order rate constant obtained from exponential fitting (error bars from 95% confidence intervals): $k_{obs} = 0.014 \pm 0.002$ min^{-1} at 283K.

4. Computations

The PBE1PBE[9] functional (ultrafine integration grid), def2-SVP basis set,[10] and conductor-like polarizable continuum model (CPCM)[11] for toluene were used for geometry optimizations and vibrational frequency characterization in Gaussian 16.[12] Single-point energies were calculated with M06[13]/def2-TZVPD using ORCA.[14] CCSD(T) calculations were executed in ORCA. 3D structures presented in the main manuscript were created using CYLview.[15]

Al-water O-H bond energy

Both PBE1PBE and M06 suggest that, upon coordination, the covalent water bond is weakened to less than 15 kcal/mol on the potential energy surface (ΔE). To evaluate whether this bond energy estimate is an overestimate or underestimate, we carried out CCSD(T) calculations with both the def2-SVP and def2-TZVP basis sets on a model system that replaced 2,6-di-*iso*-propyl groups with methyl groups. Importantly, the CCSD(T) method suggests the PBE1PBE and M06 values are likely overestimates.

$$\dot{\text{L}}\text{-Al(Me)(H}_2\text{O)} \longrightarrow \text{L-Al(Me)(OH)} + \text{H}^{\bullet}$$

**Method**	**$\Delta E/\Delta H/\Delta G$ (kcal/mol)**
PBE1PBE/def2-SVP<br>(calculated in Gaussian)	14.5/9.0/2.8
M06/def2-TZVPD//PBE1PBE/def2-SVP<br>(calculated in ORCA)	12.7/8.1/1.0
CCSD(T)/def2-SVP//PBE1PBE/def2-SVP<br>(calculated in ORCA)	5.8/1.5/-5.1<br>(model system; enthalpy and Gibbs energy corrections were added using PBE1PBE/def2-SVP values)
CCSD(T)/def2-TZVP//PBE1PBE/def2-SVP<br>(calculated in ORCA)	5.8/1.6/-5.0<br>(model system; enthalpy and Gibbs energy corrections were added using PBE1PBE/def2-SVP values)

Understanding the low O-H bond energy

The driving force for the very weak O-H bond can be understood using equations B-E shown below (M06/def2-TZVPD//PBE1PBE/def2-SVP). These equations indicate that a major

driving force is the one electron reduction of the Fe radical species, which is exothermic by 23 kcal/mol. The Al-water complex acidity is only moderately lowered compared to water, with a proton affinity of 320 kcal/mol (equation C). Equation D shows that the ligand holds the electron with a detachment energy of 102 kcal/mol. Lastly, with electron transfer to the Fe metal center, the water acidity drops by 95 kcal/mol.

A) $\dot{\mathrm{L}}\text{-Al(Me)(H}_2\text{O)} + \cdot\mathrm{Fe} \longrightarrow \text{L-Al(Me)(OH)} + \text{H-Fe}$

B) $\cdot\mathrm{Fe} + 1e \longrightarrow :\mathrm{Fe}^{\ominus}$

C) $\dot{\mathrm{L}}\text{-Al(Me)(H}_2\text{O)} \longrightarrow \text{L-Al(Me)(OH)} + \mathrm{H}^{\oplus}$

D) $\text{L-Al(Me)(H}_2\text{O)} \longrightarrow \mathrm{L}^{\oplus}\text{-Al(Me)(H}_2\text{O)} + 1e$

E) $\mathrm{L}^{\oplus}\text{-Al(Me)(H}_2\text{O)} \longrightarrow \text{L-Al(Me)(OH)} + \mathrm{H}^{\oplus}$

**Reactions**	**Δ*E***	**Δ*H***	**Δ*G***
**A**	-49.1	-49.6	-47.7
**B**	-22.9	-23.5	-23.8
**C**	320.5	313.0	306.2
**D**	101.7	103.9	103.5
**E**	224.7	217.9	211.6

Comment on one-step water addition transition state

Based on experimental rate measurements it was proposed that water (and alcohol) is not involved in (or before) the rate limiting step for reaction with the Al-Fe complex. Therefore, it is perhaps surprising that we did locate a one-step transition state for water addition across the Al-Fe bond, and the barrier is relatively low. The enthalpy for this transition state is 9 kcal/mol and the Gibbs energy is 20 kcal/mol. The structure for this transition state can be found with the other xyz coordinates. This indicates that DFT methods likely have a very difficult time estimating barrier heights and relative energies for metal-metal bond cleavage reactions.

References

1. Borys, A. M. An Illustrated Guide to Schlenk Line Techniques. *Organometallics* **42**, 182–196 (2023).
2. Pangborn, A. B., Giardello, M. A., Grubbs, R. H., Rosen, R. K. & Timmers, F. J. Safe and Convenient Procedure for Solvent Purification. *Organometallics* **15**, 1518–1520 (1996).
3. Sinhababu, S., Radzhabov, M. R., Telser, J. & Mankad, N. P. Cooperative Activation of CO_2 and Epoxide by a Heterobinuclear Al–Fe Complex via Radical Pair Mechanisms. *J. Am. Chem. Soc.* **144**, 3210–3221 (2022).
4. Caise, A. *et al.* On the Viability of Catalytic Turnover via Al−O/B−H Metathesis: The Reactivity of β-Diketiminate Aluminium Hydrides towards CO2 and Boranes. *Chem. – Eur. J.* **24**, 13624–13635 (2018).
5. Chin, T. T. *et al.* Dicarbonyl(η5-Cyclopentadienyl)Nitrosyl Complexes of Chromium, Molybdenum, and Tungsten. in *Inorganic Syntheses* 196–198 (John Wiley & Sons, Ltd, 1990). doi:10.1002/9780470132593.ch50.
6. Müller, P. Practical suggestions for better crystal structures. *Crystallogr. Rev.* **15**, 57–83 (2009).
7. Sheldrick, G. M. A short history of SHELX. *Acta Crystallogr. A* **64**, 112–122 (2008).
8. Bai, G., Singh, S., Roesky, H. W., Noltemeyer, M. & Schmidt, H.-G. Mononuclear Aluminum Hydroxide for the Design of Well-Defined Homogeneous Catalysts. *J. Am. Chem. Soc.* **127**, 3449–3455 (2005).
9. Perdew, J. P., Burke, K. & Ernzerhof, M. Generalized Gradient Approximation Made Simple. *Phys. Rev. Lett.* **77**, 3865–3868 (1996).
10. Weigend, F. & Ahlrichs, R. Balanced basis sets of split valence, triple zeta valence and quadruple zeta valence quality for H to Rn: Design and assessment of accuracy. *Phys. Chem. Chem. Phys.* **7**, 3297 (2005).
11. Tomasi, J., Mennucci, B. & Cammi, R. Quantum Mechanical Continuum Solvation Models. *Chem. Rev.* **105**, 2999–3094 (2005).
12. Frisch, M. J.; Trucks, G. W.; Schlegel, H. B.; Scuseria, G. E.; Robb, M. A.; Cheeseman, J. R.; Scalmani, G.; Barone, V.; Petersson, G. A.; Nakatsuji, H.; Li, X.; Caricato, M.; Marenich, A. V.; Bloino, J.; Janesko, B. G.; Gomperts, R.; Mennucci, B.; Hratchian, H. P.; Ortiz, J. V.; Izmaylov, A. F.; Sonnenberg, J. L.; Williams-Young, D.; Ding, F.; Lipparini, F.; Egidi, F.; Goings, J.; Peng, B.; Petrone, A.; Henderson, T.; Ranasinghe, D.; Zakrzewski, V. G.; Gao, J.; Rega, N.; Zheng, G.; Liang, W.; Hada, M.; Ehara, M.; Toyota, K.; Fukuda, R.; Hasegawa, J.; Ishida, M.; Nakajima, T.; Honda, Y.; Kitao, O.; Nakai, H.; Vreven, T.; Throssell, K.; Montgomery, Jr., J. A.; Peralta, J. E.; Ogliaro, F.; Bearpark, M. J.; Heyd, J. J.; Brothers, E. N.; Kudin, K. N.; Staroverov, V. N.; Keith, T. A.; Kobayashi, R.; Normand, J.; Raghavachari, K.; Rendell, A. P.; Burant, J. C.; Iyengar, S. S.; Tomasi, J.; Cossi, M.; Millam, J. M.; Klene, M.; Adamo, C.; Cammi, R.; Ochterski, J. W.; Martin, R. L.; Morokuma, K.; Farkas, O.; Foresman, J. B.; Fox, D. J. Gaussian 16, revision B.01; Gaussian, Inc.: Wallingford CT, 2016.

13. Zhao, Y. & Truhlar, D. G. The M06 suite of density functionals for main group thermochemistry, thermochemical kinetics, noncovalent interactions, excited states, and transition elements: two new functionals and systematic testing of four M06-class functionals and 12 other functionals. *Theor. Chem. Acc.* **120**, 215–241 (2008).
14. Neese, F. Software update: The ORCA program system—Version 5.0. *WIREs Comput. Mol. Sci.* **12**, e1606 (2022).
15. Legault, C. Y. CYLview20. (2020).
